# Understanding multilevel barriers to childhood vaccination uptake among Internally Displaced Populations (IDPs) in Mogadishu, Somalia: a qualitative study

**DOI:** 10.1186/s12889-023-16153-1

**Published:** 2023-10-17

**Authors:** Mohamed Jelle, Andrew J Seal, Hodan Mohamed, Hani Mohamed, Mohamed Sheikh Omar, Sadik Mohamed, Amina Mohamed, Joanna Morrison

**Affiliations:** 1 UCL Institute for Global Health, London, UK; 2 Action Against Hunger (ACF), Mogadishu, Somalia; 3 Independent Consultant, London, UK

**Keywords:** Vaccination coverage, Measles, PLA interventions, Somalia, Internally displaced persons (IDPs), Children

## Abstract

**Background:**

Disparities in vaccination coverage exist in Somalia with Internally Displaced Persons (IDPs) being among the groups with the lowest coverage. We implemented an adapted Participatory Learning and Action (PLA) intervention, which focused on routine vaccinations among displaced populations living in Mogadishu IDP camps. The intervention was successful in improving maternal knowledge and vaccination coverage but unsuccessful in improving timely vaccination. We conducted a qualitative study to understand this result and analyze the multi-level barriers to routine childhood immunization uptake.

**Method:**

In this qualitative study we used observation data from 40 PLA group discussions with female caregivers and purposively sampled nine vaccination service providers and six policy makers for interview. We also reviewed national-level vaccine policy documents and assessed the quality of health facilities in the study area. We used the socioecological framework to structure our analysis and analyzed the data in NVivo.

**Results:**

The barriers to childhood vaccination among IDPs at the individual level were fear due to lack of knowledge, mistrust of vaccines, concerns about side effects and misinformation; opportunity costs; and costs of transportation. At the interpersonal level, family members played an important role as did the extent of decision-making autonomy. Community factors such as cultural practices, gender roles, and household evictions influenced vaccination. Organizational issues at health facilities such as waiting times, vaccine stock-outs, distance to the facility, language differences, and hesitancy of health workers to open multi-dose vials affected vaccination. At the policy level, confusion about the eligible age for routine vaccination and age restrictions for catch-up vaccination and certain antigens such as BCG were important barriers.

**Conclusion:**

Complex and interrelated factors affect childhood vaccination uptake among IDPs in Somalia. Interventions that address multiple barriers simultaneously will have the greatest impact given the complex nature of vulnerabilities in this population. There is a need to strengthen the health system and connect it with existing community structures to increase demand for services. Our research highlights the importance of formative research before implementing interventions. Further research on the integration of health service strengthening with PLA to improve childhood vaccination among IDPs is recommended.

**Trial registration number:**

ISRCTN-83,172,390. Date of registration: 03/08/2021.

## Introduction

It is estimated that vaccines prevent about 2–3 million deaths every year by reducing incidence of diseases like diphtheria, tetanus, pertussis (whooping cough), influenza, and measles. They also protect the unvaccinated by conferring herd immunity [1, 2]. Despite this potential, vaccination coverage is very low in many parts of the world, including countries in Africa [3] due to demand and supply issues[4]. In 2019, it was estimated that over 71% of the 19.7 million children under 5 years old that had received zero doses of vaccine lived in Africa and in countries affected by conflicts [5, 6].

Somalia has experienced over three decades of political turmoil, protracted armed conflict, and intermittent natural disasters. Currently, there are about 2.9 million people (19% of the population) in Internally Displaced Persons (IDP) camps. This is in contrast to other countries in the region which have experienced conflict such as Ethiopia where only 1.8% of the population are in IDP camps or South Sudan where 12.7% of the population are in IDP camps[7, 8]. Most IDPs in Somalia are from marginalized or minority clans [9, 10], which makes them more vulnerable to forced evictions, poor housing conditions, compulsory relocations, and social and political exclusion than the more powerful clans [11, 12]. None of the IDP camps have formal status, and there are no government or UN-operated IDP “camps” that can provide assured shelter, security, and services [13].

### Childhood vaccination in Somalia

The Expanded Program on Immunization (EPI) policy in Somalia, launched in 1978, provides free vaccination for all children < 24 months of age against vaccine-preventable diseases [14]. It involves administrating one dose of Bacillus Calmette–Guérin (BCG) and one dose of Oral Poliovirus Vaccines (OPV) at birth, three doses of DPT-HepB-Hib (Penta) and OPV vaccines at 6, 10 and 14 weeks of age, one dose of measles and inactivated poliovirus vaccine (IPV) vaccine at 9 months and second dose of measles 2 and IPV 2 at 15 months old. The policy recommends that all children should complete the primary vaccination schedule by their first birthday [15]. Children who have not completed the primary immunization schedule by their first birthday are eligible to be vaccinated up to the age of two years. Unvaccinated children older than one year are recommended to receive OPV, IPV, Pentavalent & MCV. Supplementary vaccinations are recommended such as polio and /or measles doses be given irrespective of the child’s history of vaccination. To reduce missed opportunities, the policy emphasises that all health facilities offer vaccination services as frequently as possible, according to the vaccination schedules. They should vaccinate a child whenever there is an opportunity, even if this requires opening a multi-dose vial for one child.

Despite this guidance, only 30–40% of children have been fully vaccinated [16] and vaccine-preventable diseases (VPD) are prevalent in Somalia [17]. Measles is a leading cause of death in children under the age of five. Large-scale outbreaks have caused excess mortality during both the 2011 famine and the 2017 food crisis [18]. Disparities in vaccination coverage exist between the provinces, and between urban and rural areas. In Mogadishu, the most populous city of Somalia, there is 49% coverage in the IDP camps as compared to 59% in other urban areas [19]. Previous studies in Somalia have found that low vaccination uptake was influenced by opportunity and non-opportunity costs, lack of awareness, rumors and misinformation, cultural beliefs, and low trust in vaccines. Risk factors for non-vaccination were related to the old age of the caregiver, low education level of fathers, low household income, and living in hard-to-reach areas. In addition, harmful gender norms restricting autonomy and movement often prevented female caregivers from vaccinating children [20, 21].

Community participation to improve health outcomes is being increasingly promoted by humanitarian actors to support the uptake of interventions in humanitarian contexts [22], but there is limited evidence of the effectiveness of participatory approaches in improving vaccination uptake among IDPs. Women’s groups using a participatory learning and action (PLA) approach have been widely used in the field of public health and have shown potential to address health-related challenges in low-resource settings especially in contexts not experiencing humanitarian emergencies [23–25]. They were also shown to be cost-effective in low-resource settings [23, 26]. PLA enables communities to come to a collective understanding of the drivers of health problems, and work together to address them [27]. Research in high-income countries has used PLA to work with hard-to-reach immigrants [28, 29]. The success of PLA and the need for an intervention to address the complex determinants of vaccination uptake led us to develop an adapted PLA intervention, which was implemented in partnership with local women’s groups. The ‘Improving Vaccination Awareness & Coverage in Somalia (IVACS)’ study evaluated whether this intervention could improve vaccination uptake among children living in IDP camps.

### Abaay-Abaay groups (AAG)

AAG also called “Abay Siti” are all-female community groups that are found in many parts of Somalia. They are spontaneously formed by interested women and have traditionally focused on blending Somali custom with religious activities, typically centered around supporting each other and their communities. The groups are headed by an elderly woman known as Khalifada, who should be familiar with local history and Islamic teaching. She should be well-known as a devout Muslim sister who fears God [30]. Other attributes include her perceived leadership ability, and ability to provide exemplary childcare. Groups are commonly found in stressed and displaced populations, including most IDP camps in Mogadishu. Most members are from the same clan, village, or district and live within the same IDP camp or group of camps. Groups provide a safe space where women come together to support one another emotionally and share their challenges. They are also a platform where young and new mothers can gain knowledge from older and experienced women. During meetings, women may discuss their problems and request the group to pray for them. Pregnant women may also request prayers for a safe delivery.

### The PLA intervention

After obtaining an invitation from each Khalifada, a trained facilitator guided weekly AAG group participants through an adapted four-phase PLA cycle. Based on our experience of conducting research in humanitarian contexts and our formative research, we adapted the facilitator manual developed by Women and Children First (UK)[31]. We ran 40 groups in 5 camps for a period of two months (from 27/07/2021 to 29/09/2021). Groups were pre-existing but open to all women. Women of reproductive age and adolescent girls, particularly those who were pregnant or had children were encouraged to attend. An average of 38 women attended each meeting. Groups were given a one-time distribution of goods worth $108 to help with the continuation of the meetings. Each group got 30 kg of beans, 2.5 kg of popcorn, 5 L of sesame oil, 1 kg of tea leaves, 25 kg of sugar and 40 kg of maize.

Groups used participatory methods such as locally developed picture cards, games, and stories to identify and prioritize child health problems for under-5 children. They identified 9–16 diseases and then voted to prioritize three diseases. In all camps, measles and pneumonia were prioritized. Other diseases selected included pertussis, scabies, malnutrition, skin diseases, and cholera. They then developed and implemented locally available strategies to address those child health problems. There was a onetime stakeholders’ meeting where group members met with vaccination service providers from NGOs to discuss their progress on addressing barriers and the challenges that they faced while seeking health services. As a result of this meeting, service providers extended the scope of their mobile vaccination teams. Strategies chosen by the AAG were awareness raising through household visits, encouraging caregivers to take their children for vaccination on vaccination days, taking children of working mothers to vaccination centers, and community cleaning. Facilitators visited some households to understand what caregivers were learning from group members’ home visits and encourage further interaction where needed. Groups then evaluated their strategies, reflecting on their progress in addressing common child health problems.

### Theory of change

We hypothesized that the PLA intervention would increase vaccination uptake through: (1) providing an opportunity for group members to learn about vaccination types, schedules, and benefits; (2) encouraging interaction in communities about routine vaccination and its’ benefits; and (3) facilitating discussion between IDPs and vaccination service providers about the challenges of accessing services that would encourage action to address these.

### The trial

We conducted a cluster-randomized controlled trial in 10 camps for internally displaced persons (IDPs) near Mogadishu. The purpose was to assess the effectiveness of AAG with PLA compared to AAG alone (control) in improving primary outcomes, including measles vaccination coverage, completion of the Penta vaccine series, timely vaccination, and maternal knowledge of child health. The secondary outcome was possession of a health record cards.

The results showed that the PLA intervention had a positive impact. Maternal/caregiver knowledge scores increased by 7.9 points (out of a maximum of 21) compared to the control group. Measles vaccination coverage and completion of the pentavalent vaccine series also improved significantly. However, there was no significant improvement in adherence to timely vaccination. On the other hand, the possession of a home-based child health record card increased from 18 to 35% in the intervention group [32]. A summary of the main findings from the trial is shown in Table 1.

**Table 1:** PLA intervention effects on primary and secondary outcomes

		Adjusted prevalence or mean score (95% CI)								Adjusted Regression Model			
		Control				hPLA							
Trial arm		Baseline		Endline		Baseline		Endline		aOR or Score		(*95% CI)*	*P-value*
**Primary outcomes**													
Caregiver knowledge score^1^		7.79	(6.66, 8.92)	8.02	(6.76, 9.27)	8.24	(7.33, 9.14)	16.36	(15.73, 17.00)	7.89		(6.93, 8.85)	**< 0.0001**
Measles vaccination (%)^2^		63.6	(52.4, 75.0)	69.8	(59.2, 75.5)	67.9	(60.3, 75.5)	86.6	(80.5, 92.7)	2.43		(1.96, 3.01)	**< 0.0001**
Penta series completion (%)^3^		62.2	(54.2, 73.0)	68.0	(57.7, 78.3)	65.4	(54.4, 76.4)	85.4	(76.7, 94.0)	2.45		(1.27, 4.74)	**0.008**
Timely EPI vaccination (%)^4^		39.7	(28.1, 51.4)	37.5	(29.0, 46.0)	39.7	(29.0, 46.0)	40.0	(15.1, 64.9)	1.12		(0.39, 3.26)	0.828
Vaccination preference (%)^5^		95.8	(91.4, 100)	96.4	(93.6, 99.1)	95.2	(91.3, 99.1)	99.4	(98.4, 100)	2.30		(-0.33, 4.93)	0.086
**Secondary outcome**													
Child health record card (%)^4^		18.9	(8.9, 28.8)	21.3	(18.5, 24.1)	18.0	(10.7, 25.3)	35.3	(28.6, 42.0)	2.86		(1.35, 6.06)	**0.006**

In all analyses, except caregiver knowledge and vaccination preference, results are adjusted for child age and sex, and the baseline

value of the outcome variable. For caregiver knowledge and vaccination preference results were adjusted for age, educational status, previous vaccination of the caregiver, and baseline responses

^1^ We assessed impact in mothers/caregivers using linear regression and we report the adjusted regression coefficient. Possible scores ranged from 0–21

^2^ We assessed impact in children aged 9–59 months using logistic regression and we report the adjusted odds ratio

^3^ Impact was assessed in children aged 12–27 months using logistic regression and we report the adjusted odds ratio

^4^ We assessed impact in children aged 0–59 months using logistic regression and we report the adjusted odds ratio

^5^ We assessed impact in mothers/caregivers using logistic regression and we report the adjusted odds ratio

We present findings from process evaluation research that was conducted before and during the PLA intervention. We used a socioecological framework to analyze the multi-level barriers to timely childhood vaccination.

## Methods

### Study setting

The study was conducted in IDP camps located in the Afgooye Corridor in Kahda district, Mogadishu. Kahda is one of the main districts of Banadir region that hosts most of the IDPs in Mogadishu [33]. Of the 2.9 million IDPs in Somalia, over 2 Million are in South Central, of which 25% live in Mogadishu [34]. The majority of the IDPs settled in informal, sub-standard IDP settlements on private lands [34, 35]. They are managed by informal settlement managers, also known as gatekeepers who provide land, security, conflict management and social services such as assistance with burial and birth arrangements in exchange for payment in either cash or in kind [13]. The camps are often overcrowded, lack basic water, sanitation, and health services. Most households have about six members. Early marriage is common and age at first childbirth is around 17 years. Illiteracy among caregivers is around 90% [36].

Most IDPs in these camps are from marginalized or minority clans from Bay, Bakool, or the Shabelle regions [9, 10]. Only about 10% of caregivers are in paid work. The average monthly household income is about $68 [36]. Free essential health services, including vaccination, are provided by non-governmental organizations (NGOs) with support from the Government and UN agencies at fixed healthcare facilities and mobile clinics [21]. Community health workers (CHWs) conduct outreach services for vaccination and nutrition programs.

### Ethics statement

The Ministry of Health & Human Services of the Federal Republic of Somalia (reference: MOH&HS/DGO/0381/Feb/2021) and the UCL Research Ethics Committee of UCL (project ID: 4684/003) granted ethical approval for the study. The trial was registered on 3rd August 2021 with ISRCTN (ISRCTN83172390). Informed verbal consent was obtained from camp leaders in all IDP camps before starting data collection and informed verbal and written consent was also obtained from participants.

### Sampling

All five health facilities from the study area were sampled for a health facility assessment to describe the availability and quality of vaccination services. A list of all five health facilities and their Global Positioning System (GPS) coordinates was first obtained from the health and nutrition teams of Action Against Hunger (ACF), which was confirmed during the initial mapping to identify clusters in the study area. We asked members of the IDP communities which health facilities they used and triangulated information from the list provided by ACF. Of the five health facilities, three were run by INGOs and two by local NGOs, and all provided free primary health care.

Qualitative purposive sampling was used with three different types of participants: (1) female caregivers who had participated in the PLA groups from all five intervention camps, (2) health workers who provided vaccination in the study area and (3) Policy influencers such as NGO, UN or Government workers who were responsible for vaccination programming and/or policymaking. Four policy influencers were sampled following discussions with the Health and Nutrition team of our operational partner, ACF who has been implementing integrated humanitarian assistance programmes in the Afgooye Corridor for the past 28 years. Snowball sampling was then used to identify a further two policy influencers. Participants were approached at their place of work or at home and invited to participate.

### Data collection

#### Interviews

We conducted nine semi-structured interviews (SSI) with health workers and six key informant interviews (KII) with policy influencers to explore the multilevel barriers to childhood vaccination uptake among IDPs. We developed separate topic guides for SSIs and KIIs, but both were informed by the existing literature on barriers to vaccination uptake.

SSIs were conducted in-person in November 2021 at the health facilities to explore health system level barriers to vaccination. They were undertaken in Somali by the third author (HM), who was trained and supervised by the first author (MJ) who is a Somali speaking qualitative researcher. KIIs were conducted in English (as all participants were fluent English speakers) online from May to December 2021 by MJ using Microsoft Teams to explore policy level barriers affecting vaccination uptake.

#### Observations

Two female group facilitators ran the PLA intervention and collected data from groups between July and September 2021. The facilitators were locally recruited and had completed university education. They were recruited after an assessment of their communication skills, motivation, familiarity with the study areas, and language skills. They received one week’s training about the PLA approach, group facilitation, and common vaccine preventable diseases, their prevention and control, vaccination schedule, types of routines vaccines and benefits of vaccination. They were given training on how to write structured reports after each meeting. Facilitators were supervised by a research officer (HM), who was a medical doctor with previous health research and programming experience in the area and lived in Mogadishu.

Facilitators wrote structured reports of each group meeting in English. Reports contained information on common childhood vaccine preventable diseases, the main barriers to vaccination uptake and how they addressed these barriers.

#### Literature review of policy documents

A literature search to identify vaccination policy documents was conducted to understand the policy context within which the intervention was run. Inclusion criteria were national-level immunization policies, immunization strategy documents and vaccination specific guideline/summary documents in Somalia. Internet searches using the terms: ‘immunization’ AND ‘policy’ OR ‘guideline’ AND ‘Somalia.’ conducted from April to December 2021 yielded four policy documents for review. These documents addressed vaccination goals, guidelines and provided information to stakeholders and communities about the public health benefits about vaccination and target populations. The documents were published by either the Ministry of Health & Human Services (MoHHS) or the World Health Organization (WHO) between 2014 and 2020. All documents were publicly available in English. Of the four documents, two were policy documents, one was a strategy document and one a summary of EPI target achievements.

#### Health facility assessment

We used a locally developed rapid health assessment tool. This measured some key indicators and provided a scorecard of vaccination services at each health facility. It described potential bottlenecks to high quality and accessible service delivery, including geographical location, supply chain, human resources capacity, cold chain capacity, availability of health cards, frequency of stock-outs, and the frequency of vaccination service availability.

### Data analysis

The socio-ecological model (SEM) was used to structure the analysis. We used this framework because it captures the complexity of influences on vaccination behavior. The SEM acknowledges that a person’s behavior is influenced by five over-arching domains: individual, interpersonal, community, organizational and policy. The primary principles of the SEM are that individual behaviors both influence and are influenced by multilevel factors and understanding these factors and their interaction is necessary for the successful uptake of interventions [1]. The SEM is increasingly being used to study multilevel barriers to immunization uptake in low and middle-income contexts [1, 37]. We used the domains of SEM to structure the analysis of all data sources.

MJ reviewed the observation reports while the intervention was running so that he could discuss the data with facilitators and note interesting and recurrent themes. Data from health worker and policy influencer interviews were digitally recorded and transcribed verbatim into English. MJ compared five randomly chosen SSI recordings with English transcripts to check the quality of translation.

Policy documents; observation reports, data from the health facility assessments and interview transcripts were imported into NVivo 12 for analysis [1]. First, MJ read seven transcripts, eight observation reports and all five-health facility assessment reports. He then developed codes about what affected uptake of vaccination under each level of the SEM: individual barriers; interpersonal barriers, community barriers, organizational barriers; and policy barriers. All policy documents were also reviewed to compare policy guidelines with reported common practices in the health facilities. MJ discussed the initial findings with the research team and finalized codes to use under each level of the SEM. MJ then analyzed all the data using NVivo version 12 [38].

## Results

**Fig. 1:**
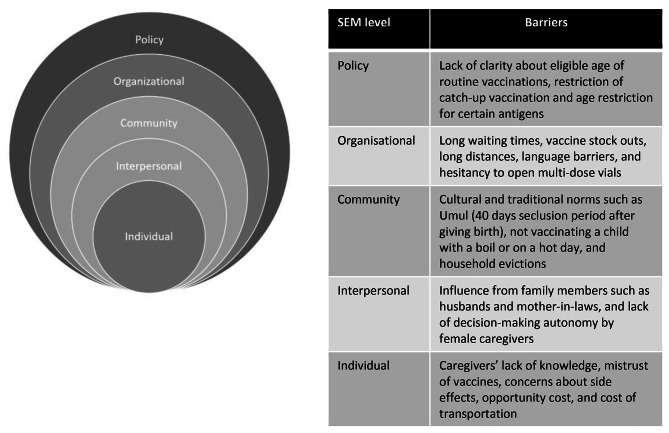
Barriers to vaccination uptake

We present our findings on the barriers to childhood vaccination uptake from the individual to the policy level (Fig. 1).

### Individual level barriers

Most female caregivers were aware about the importance of routine vaccinations and knew that vaccination prevents diseases like measles. However, they did not know about the types of vaccines, schedules, the possibility of administering multiple vaccines at the same time and the existence of vaccines that protects children from multiple diseases such as Penta. Many could not read the health record cards of their children and the only way they could identify what kinds of vaccines children were given was by remembering the body parts through which those vaccines were administered.

*My children were vaccinated, but I do not know the names of vaccines given and when they were given. They were given on the arms and thighs. The last one was given on the thigh. (IDP women, GD).*

Fear about vaccine side effects, beliefs that vaccines may cause disease, and doubts about vaccination safety were reported as important barriers, which prevented childhood vaccination.

*I think some parents - whose children experienced side effects or had met with a child who got swelling or fever after vaccination - won’t take their children for vaccination because of the fear of their children getting sick (Health worker, interview)*

Opportunity costs were also reported as an important barrier to vaccination especially for female-headed families who depended on daily wage labor for their livelihood. These caregivers found it difficult to afford the time to take their children to the health facility.

*Single mothers who do not have livelihoods tend to go for casual labor, which makes them miss the opportunity to come to the health centre (Health worker, interview).*

Another barrier reported by both the health workers and group discussants were the transport costs for IDPs who lived far from health facilities. They either had to walk long distances or meet the costs of local transport to vaccinate their children.

Health workers also reported that many mothers preferred traditional ways of giving birth and therefore delivered at home. This led to their children missing at-birth vaccines such as BCG and OPV.

### Interpersonal-level barriers

Group discussions revealed that caregivers often had little power to take decisions on child vaccination. Many caregivers reported that they could not have children vaccinated if their husbands or mothers-in-law were not supportive. Caregivers were also less likely to vaccinate their child if community members had negative attitudes towards vaccination.

*My husband and I have to discuss about the health of our children but if he refuses then, I can’t take the child to the centre for vaccination. (IDP woman, GD)*

### Community level barriers

Discussions with participants also revealed the existence of some cultural and traditional beliefs that may have impeded vaccination uptake. Some caregivers believed that vaccination should only happen after the 40th day after delivery, when mothers have finished their seclusion period. This period is called Umul, which is the time after the birth when extended family members come to help the new mother so that she can rest and heal. This usually resulted in children missing BCG and OPV0, which would normally be given at birth.

Some caregivers and family members believed that children should not be vaccinated around midday when the sun was hot. While others believed that a child who had a boil should not be vaccinated. This was because they believed that a child who is vaccinated when it is too hot or with a boil may be at risk of death.

Health workers and IDPs said that eviction affected vaccination uptake. It was common for IDPs to be evicted from camps near health facilities because they were usually on private land. The camps they resettle in were often far from facilities and parents had to use transport or walk long distances to vaccinate their children.

*Eviction is one of the main reasons (why parents don’t bring their children for vaccination). Even if you call the mother, she will tell you that she was evicted to a place far away and can’t reach the health centre (Health worker, interview).*

### Health system barriers

Of the five health facilities that we assessed, two local NGO-run facilities were experiencing vaccine stock-outs and were not providing any vaccination services. Of the three remaining health facilities, two experienced regular monthly stock-outs and one reported that they experienced stock-outs every few months. BCG, Penta, and measles vaccines were commonly in short supply. There were long waiting times to receive vaccinations because of staff shortages and a shortage of vaccines and health facilities able to vaccinate. This meant that sometimes parents would return from the health facility without having had their child vaccinated. In one of the health facilities, there was no vaccinator present. Only one of the five humanitarian agencies had a mobile team, which limited their ability to reach IDPs that were far from health facilities.

Another barrier to vaccination was the reluctance of health workers to open multi-dose vials unless there were enough eligible children to use all the contents of the open vials. When this happened, parents were told to return on specific days for vaccinations or their telephone numbers were recorded so they could be called to the health facility when there were enough children to vaccinate. Both health workers and policy influencers reported that BCG vials had 20 doses and they were not allowed to waste more than 50% of the doses. All health facilities reported that they provided BCG and measles only once or twice a week.

*The EPI policy recommends that if a child comes for BCG or any other vaccine, we must take this opportunity to vaccinate them but in reality, this policy is not practiced. Health workers are more concerned about the level of wastage of the vaccine, which should be less than 50%. So, they need to make sure to have 50% of the children before they can open a vial. If it’s one or two children, they don’t open due to fear of high wastage rate of the vaccine (Policy influencer, interview).*

Language barriers to vaccination existed for those that did not speak the common Mahatiri and Maay dialects. IDPs from clans such as the Jiido and Garre speak unique dialects, which most health workers did not understand. One of the health workers said:

*Many times I have met people whose dialects I couldn’t understand. When there were people with different dialects, we had colleagues who could understand those dialects and sometimes we called the camp leaders for translation (Health worker, interview).*

Health workers’ rude attitudes towards caregivers and forced vaccination were also discussed as reasons that caregivers did not use the health facilities. Some of the health workers said that they forced mothers to vaccinate their children by confiscating their children’s health card and/or denying other services like nutrition treatment. Health workers believed that this was an effective strategy to make mothers vaccinate their children.

*Some mothers, who come for the Outpatient Therapeutic feeding Program (OTP) services, may refuse their children to be vaccinated. I would ask such a mother to sit down, take the card from her and put it under the register. Then we talk. I would tell her that I would not give her card back until her child is vaccinated. If she still says ‘no’, then I would tell her that her child would not get biscuit (Plumpy’nut/Plumpy’Sup). If I would refuse to give, even the health facility manager would not be able to give it to her. Then I would ask: “wouldn’t you be worried about your malnourished child and the biscuits that they would miss?” If she couldn’t decide herself and wanted to call her husband, I would call him and would put him on loudspeaker so that she could hear what he would say (Health worker, interview).*

### Policy level barriers

Discussions with health workers and policy influencers highlighted a discrepancy between policy and practice concerning the age limit for routine vaccination. The 2020 EPI policy clearly states that children under two years old who have not completed the primary series by their first birthday are still eligible to be vaccinated. In practice, that does not happen and only children below 12 months old are vaccinated.

*The recommendation to vaccinate all children below 24 months is there but for immunization programs, it is important to make sure that a child receives all the primary vaccines before they reach one year. So, we are not applying that (recommended) policy yet. (This is happening) not only in Somalia but across many countries (Policy influencer, interview).*

Another discrepancy exists in catch-up vaccination of unvaccinated children older than 12 months. The EPI policy recommends that unvaccinated children older than one year old should receive OPV, IPV, Penta & MCV, but some health workers and policy influencers said that unvaccinated children older than 12 months were only eligible for measles and/or Polio vaccinations.

The upper age limit for catch-up vaccinations was also not in line with WHO guidelines because it limits the use of catch-up of routine vaccinations to children below 24 months of age. Whilst the local EPI policy recognizes that vaccination in emergency requires innovative solutions and flexibility, in practice, this did not extend to increasing the age limit for vaccination to older children up to five years old.

Another age-related barrier was age restriction for certain antigens. The current policy does not allow for children above 12 months to get the BCG antigen.

## Discussion

We used data from multiple sources to understand the barriers to child vaccination uptake in the context of a cluster-randomized controlled trial of PLA in urban Mogadishu IDP camps. IDP children’s access to routine vaccination was influenced by policy, the health system, and the social context. At the policy level, two key issues were salient (1) the dissonance between the Somali EPI policy and health worker practice, and (2) a lack of flexibility in applying the policy about the upper age limit for vaccination. At the health systems level, vaccine supply, human resources, and infrastructure related barriers were reported. At the community level a culture of suspicion of vaccinations and a gendered lack of autonomy for female caregivers were reported. There was evidence of health workers coercion of caregivers to vaccinate their children, which discouraged health care-seeking. Our findings are consistent with other studies on the barriers to vaccination uptake in Somalia [20, 21] but few have examined these barriers within the context of health policy and health systems in Somalia.

### Addressing policy and health system issues in context

A global systematic review and meta-analysis in 2015 found that interventions addressing demand-side barriers to vaccination may hold the greatest promise to reach underserved populations. The review also concluded that interventions based on education or knowledge transfer were generally more successful than those based on incentives [39]. Our study shows that it is important to address both demand and supply side issues in interventions. The PLA intervention largely focused on addressing demand side barriers to vaccination, and groups were unable to address many health systems and health policy issues.

PLA improved maternal knowledge and overall vaccination coverage but there was no change in timely vaccination when comparing intervention and control areas. A context of preference for home birth and postpartum seclusion may lead to newborns missing OPV0 and BCG vaccines, which are given at birth in health facilities. BCG can be given up to one year but OPV0 must be given within 2 weeks of birth [40]. By the time the child is brought for their first vaccination after 40 days, they are already due for OPV1 and are therefore not given OPV0, this leads to the child not being vaccinated on time even if they get the rest of the vaccines in the primary series [40]. The policy needs to acknowledge this cultural context and be flexible to the needs of caregivers who may not be able to attend the health facility at the ‘right’ time.

### Implications for programming

People in humanitarian emergencies such as IDPs are at higher risk from vaccine preventable diseases[41, 42], and therefore Somalia EPI policy and practice should be brought in line with guidance from the World Health Organization (WHO) Strategic Advisory Group of Experts on Immunization (SAGE) in acute humanitarian emergencies. SAGE recommends expansion of vaccination target groups during emergencies to include older children and adults [43]. Our study findings reveal missed opportunities and age restrictions that may prevent vulnerable IDP children getting vaccinated. More research is necessary to understand the reasons why the Somali EPI policy does not follow WHO guidance. More research is also needed to understand the reasons for the dissonance between policy and practice about eligible ages for both routine and catch-up vaccination and the opening of multi-dose vials. Based on this research, Ministry of Health, WHO, UNICEF and other health actors should undertake a review of the current policy and put strategies in place to address the reasons why health workers are not practicing some aspects of the EPI policy to reduce missed opportunities for vaccination.

Mobile clinics have been used to achieve high vaccination coverage in migrant populations [44]. This study showed that forced evictions are common among IDPs and they can experience multiple evictions. This has the potential to disrupt vaccination services for already vulnerable groups. Health agencies should consider tracking evicted camps from their catchment areas and continuing vaccination through mobile teams. Mobile clinics would also enable vaccinations to reach children who were born at home or during the postpartum seclusion period.

### Inclusion of the wider family in vaccination promotion sessions

A large body of evidence demonstrates the strong link between maternal education and childhood vaccination uptake [45]. As a result, many of the strategies and interventions used to improve vaccination coverage are focused on caregivers as the primary audience. This study shows that, caregivers sometimes have limited capacity to influence and enact decisions about their child’s health, and their decisions are often influenced by community norms and beliefs. Therefore, vaccination programme designers should implement interventions that engage families and communities to improve the success of such interventions. Previous studies have shown that grandmothers and fathers are key influencers of maternal and child health and should therefore be included in interventions [46–48].

### Expanding the age limit for catch-up vaccinations

Research has shown that large cohorts of susceptible people who are unvaccinated or partially vaccinated remain among hard-to-reach populations such as IDPs; and there are benefits of vaccinating older children and adults to maintain community protections during emergencies [49]. Yet our research shows that children older than 24 months are only eligible for measles and polio vaccinations and only up to the age of five years old. Arguably, these older children are more mobile and therefore could easily spread diseases if they are not vaccinated. A review by Grais et al. on measles vaccination in humanitarian contexts found that preventable measles deaths occurred in campaigns that targeted younger children below the age 5 years. The review found that 18% of the cases occurred in children aged 5–15 years [50]. Health actors should review the current Somali EPI policy considering these findings and expand the age for catch-up routine vaccination to include children older than 24 months. The eligible age for measles vaccination should also be relaxed to include children older than five years old. Offering catch-up vaccinations to older children should be prioritized to reduce vaccination gaps of IDP children and to broaden the focus of vaccination programmes towards a life-course approach [51].

The current policy restricts eligibility for BCG vaccination to children below 12 months. This is inconsistent with WHO guidelines that recommend BCG vaccination to older age groups including unvaccinated older children, adolescents and adults from settings with high incidence of TB and/or high leprosy burden [52]. Considering the endemic nature of TB among displaced populations, MoH with support from other health actors should review the current policy to include children older than 12 months in the target group. Evidence-based approaches should be used to identify the most appropriate target groups.

### Limitations

All observations and interviews with IDPs were conducted in the Somali language but coding and final analysis were done on the translated transcripts and reports written in English. Some loss of meaning may have occurred during the translations. One of the researchers conducting this study had prior experience with vaccination programming in IDP camps, which could have biased her interview technique. The use of topic guides, triangulation with data collected by other researchers and awareness of this potential for bias helped us account for this in the analysis. Time constraints meant that we didn’t collect data after the intervention was completed. This meant that we were not able to explore stakeholder opinions on how they thought that the intervention might or might not have affected behaviors, and how successful the intervention had been at addressing gender norms and other cultural barriers.

## Conclusions

Our research has shown that there is potential for adapted PLA approaches to increase demand for vaccination services, but formative research to understand the policy and health systems barriers, as well as community level constraints to vaccination is essential to design an appropriate intervention. Our study findings suggest that IDP children’s access to routine vaccination is influenced by the policy, health system, and social context. Therefore, a PLA intervention combined with health system strengthening and policy engagement, to address both demand and supply side barriers to vaccination is necessary in this context. It is essential to address the reasons why the EPI policy isn’t fully implemented and relax the age limit for catch-up vaccination. This would allow children above 24 months to get routine vaccinations in line with the WHO guidelines. Policy and community engagement is necessary for uptake of timely vaccination and improvement in health outcomes in this vulnerable IDP population.

Our study provides three key contributions to the existing body of literature. Firstly, we emphasize the importance of conducting process evaluation research to understand how the intervention’s implementation and outcomes are influenced by the specific context and population group. Secondly, we focus on Internally Displaced Populations (IDPs) and highlight the unique challenges they face in accessing healthcare services, particularly immunization, which is an area with limited existing research. Lastly, our socio-ecological approach allows us to comprehensively analyze the factors influencing timely immunization and gain a deeper understanding of how interventions like PLA can effectively work in this context.

## Data Availability

The datasets used and/or analyzed during the current study are not publicly available. They are available from the corresponding author on reasonable request, subject to approval from the ethics committee that approved the study.

## Abbreviations

AAG: Abaay Abaay Groups
ACF: Action Against Hunger
BCG: Bacille Calmette-Guérin
CHWs: Community Health Workers
EPI: Expanded Programme on Immunization
IDPs: Internally Displaced Persons
IPV: Inactivated Poliovirus Vaccine
IVACS: Improving Vaccination Awareness & Coverage in Somalia
LMICs: Low and Middle Income Countries
MoH: Ministry of Health
MCV: Measles Containing Vaccine
OPV: Oral Polio Vaccine
PLA: Participatory Learning and Action
SEM: Socio-Ecological Model
VPD: Vaccine Preventable Disease
UCL: University College London
UCL: REC University College London Research Ethics Committee
